# Non-Destructive Detection of Pesticide-Treated Baby Leaf Lettuce During Production and Post-Harvest Storage Using Visible and Near-Infrared Spectroscopy

**DOI:** 10.3390/s24237547

**Published:** 2024-11-26

**Authors:** Dimitrios S. Kasampalis, Pavlos I. Tsouvaltzis, Anastasios S. Siomos

**Affiliations:** 1 Department of Horticulture, Aristotle University, 54124 Thessaloniki, Greece; siomos@agro.auth.gr; 2 Horticultural Sciences Department, Southwest Florida Research and Education Center, University of Florida, Immokalee, FL 34142, USA; ptsouv@ufl.edu

**Keywords:** chemometrics, antioxidant enzymes, discrimination, feature extraction, partial least squares

## Abstract

The market demand for baby leaf lettuce is constantly increasing, while safety has become one of the most important traits in determining consumer preference driven by human health hazards concerns. In this study, the performance of visible and near-infrared (vis/NIR) spectroscopy was tested in discriminating pesticide-free against pesticide-treated lettuce plants. Two commercial fungicides (mancozeb and fosetyl-al) and two insecticides (deltamethrin and imidacloprid) were applied as spray solutions at the recommended rates on baby leaf lettuce plants. Untreated-control plants were sprayed with water. Reflectance data in the wavelength range 400–2500 nm were captured on leaf samples until harvest on the 10th day upon pesticide application, as well as after 4 and 8 days during post-harvest storage at 5 °C. In addition, biochemical components in leaf tissue were also determined during storage, such as antioxidant enzymes’ activities (peroxidase [POD], catalase [CAT], and ascorbate peroxidase [APX]), along with malondialdehyde [MDA] and hydrogen peroxide [H_2_O_2_] content. Partial least square discriminant analysis (PLSDA) combined with feature-selection techniques was implemented, in order to classify baby lettuce tissue into pesticide-free or pesticide-treated ones. The genetic algorithm (GA) and the variable importance in projection (VIP) scores identified eleven distinct regions and nine specific wavelengths that exhibited the most significant effect in the detection models, with most of them in the near-infrared region of the electromagnetic spectrum. According to the results, the classification accuracy of discriminating pesticide-treated against non-treated lettuce leaves ranged from 94% to 99% in both pre-harvest and post-harvest periods. Although there were no significant differences in enzyme activities or H_2_O_2_, the MDA content in pesticide-treated tissue was greater than in untreated ones, implying that the chemical spray application probably induced a stress response in the plant that was disclosed with the reflected energy. In conclusion, vis/NIR spectroscopy appears as a promising, reliable, rapid, and non-destructive tool in distinguishing pesticide-free from pesticide-treated lettuce products.

## 1. Introduction

Baby leaf vegetables, such as lettuce, are highly valued for their rich nutritional content, which includes essential vitamins, minerals, and antioxidants. These vegetables are a vital component of a healthy diet and are usually consumed raw, increasing the importance of ensuring their safety and quality [1,2]. However, the production of baby leaf vegetables is challenging due to their short growth cycle, typically ranging from 21 to 30 days. This limited production period necessitates the careful and judicious use of pesticides to protect crops from insects and diseases while ensuring the produce remains safe for consumption [3]. Pesticides are essential in modern agricultural practices to maintain high crop yields and prevent significant losses due to pests and diseases [4]. Without pesticides, it would be nearly impossible to produce enough food to meet the demands of a growing global population, especially in the face of increasingly unpredictable climate conditions [5]. However, the use of pesticides also raises concerns about the presence of chemicals in the food supply, particularly in crops like baby leaf vegetables, which have short production periods and are consumed with minimal processing [6].

The necessity of using pesticides in vegetable production, particularly for crops like lettuce baby leaves, is underscored by the need to manage a range of pests and diseases that can devastate crops if left unchecked. However, this essential use comes with the challenge of ensuring that the residue levels on the final produce remain within safe limits set by global health authorities [7]. The short shelf life of baby leaf vegetables, exacerbated by the wounding that occurs during harvesting and handling, further complicates post-harvest management. Wounding can accelerate spoilage by increasing respiration rates and providing entry points for pathogens, thus shortening the time window for safe consumption [8].

Traditional methods for detecting pesticide residues, such as gas chromatography (GC) and liquid chromatography (LC) coupled with mass spectrometry (MS), are widely regarded as standard practice due to their high sensitivity and specificity. These methods, however, are inherently destructive, requiring extensive sample preparation that modifies the sample’s integrity. They are also time-consuming, expensive, and require highly skilled personnel and specialized equipment, which limits their adaptation for routine monitoring in large-scale agricultural operations [6,9,10].

Given these limitations, non-destructive methods have been tested as alternatives for assessing pesticide residues. Visible/near-infrared (vis/NIR) spectroscopy is a very promising non-destructive technique, offering rapid and reliable detection of pesticide residues through the interaction between light energy and the chemical constituents of the produce [9,11]. Visible and near-infrared (vis/NIR) spectroscopy has been validated as an effective non-destructive method for detecting pesticide residues across various produce types, including pak choi, purple cabbage, Hami melon, broccoli, and cauliflower. In pak choi, vis/NIR spectroscopy facilitates rapid identification of pesticide contamination due to this leafy vegetable’s high moisture content and cellular structure that enhance spectral absorption properties [12]. Purple cabbage, known for its anthocyanin-rich leaves, has shown distinct spectral responses, enabling accurate pesticide detection when using vis/NIR techniques tailored to capture pigment-related absorption peaks [13]. Hami melon, characterized by a thick outer skin, benefits from deep learning algorithms combined with vis/NIR spectroscopy, which significantly improves detection accuracy for residues on the melon surface [14]. In cruciferous vegetables like broccoli and cauliflower, vis/NIR spectroscopy has shown robust potential for pesticide detection, with spectral responses reflecting their dense cellular matrices and complex carbohydrate structures, thus enabling rapid and reliable safety assessment [15,16].

Indeed, NIR spectroscopy has been used to detect chlorpyrifos and fenvalerate insecticide residues on the surfaces of lettuce leaves one day after their application using a benchtop spectrometer in the 950–1650 nm region, providing a quick and effective way to monitor safety during both production and post-harvest stages [17].

In addition to vis/NIR spectroscopy, other non-destructive methods have shown promise in pesticide detection. Hyperspectral imaging, which captures detailed spectral information across multiple wavelengths, has been utilized to assess pesticide residues on mulberry [18], grapes [19], lettuce leaves, and other vegetables, enabling the identification and quantification of residues without altering the produce [17].

Traditional pesticide residue detection methods, such as gas chromatography (GC) and liquid chromatography (LC) coupled with mass spectrometry (MS) are highly sensitive and specific. However, these techniques require destructive sampling, lengthy preparation, and significant expense, making them impractical for high-throughput or in-field monitoring. Consequently, non-destructive techniques such as visible/near-infrared (vis/NIR) spectroscopy and surface-enhanced Raman spectroscopy (SERS) are increasingly explored as efficient alternatives for pesticide residue assessment across a range of produce, including apples, mangoes, tomatoes, olive leaves, pears, and cabbage.

For instance, SERS has been particularly effective in detecting pesticide residues on apples [20], with high sensitivity to various chemical residues on the fruit’s smooth surface, while also showing potential in quantifying residue concentrations on mangoes due to the method’s enhanced sensitivity to pigment interactions on the skin [21]. In tomatoes, SERS enables detection of even trace pesticide residues, which can be challenging due to the fruit’s high water content and smooth surface that affects spectral response [22]. For leafy samples like olive leaves, vis/NIR spectroscopy offers a quick, non-invasive way to assess pesticide residues, with the olive leaf’s surface properties amplifying spectral reflections that reveal residue presence [23].

For thicker-skinned fruits like apples and pears, laser-induced breakdown spectroscopy (LIBS) has been used successfully to detect and quantify pesticides, leveraging the fruit’s unique texture to differentiate spectral patterns [24,25]. In cabbage, LIBS and vis/NIR techniques allow for efficient pesticide residue detection on the layered leaves, with the dense leaf structure enabling distinct spectral responses that aid in distinguishing between treated and untreated samples [13]. These advancements in non-destructive testing highlight the potential of vis/NIR and SERS for routine quality control, allowing for faster and more accessible safety assessments across diverse crops.

Despite the growing application of non-destructive methods, there remains a need for more reliable and accurate techniques that can be easily integrated into routine quality-control processes during both production and post-harvest storage. The combination of vis/NIR spectroscopy with chemometrics has been shown to enhance detection capabilities, making this approach more accurate and robust in identifying pesticide residues. By analyzing complex datasets and extracting meaningful information, chemometrics can improve the reliability of vis/NIR spectroscopy for monitoring pesticide residues [14]. This combined approach is particularly beneficial for baby leaf vegetables, which require careful monitoring to ensure they remain free from harmful pesticide residues while maintaining their nutritional and sensory qualities.

In this study, we aim to apply vis/NIR spectroscopy coupled with chemometrics for the rapid detection of pesticide residues in baby lettuce leaves during production and post-harvest storage. By leveraging the non-destructive nature of this method, we seek to provide a reliable and efficient tool for ensuring that produce reaches consumers free from harmful pesticide residues, while maintaining its nutritional and sensory qualities.

## 2. Materials and Methods

### 2.1. Plant Materials

Leaf lettuce seedlings of a green romaine type cultivar (*Lactuca sativa* cv. Levistro) were purchased from a commercial planthouse 2 weeks after the seeds were sown on polystyrene trays at a plant density of 167 plants m^−2^. Plants were grown in a greenhouse at 20 °C, and one week later, two commercial fungicides, a non-systemic (mancozeb, 2.5 g/L) and a systemic fungicide (fosetyl-Al, 2 g/L), and two insecticides, a systemic (deltamethrin, 0.5 g/L) and a non-systemic one (imidacloprid, 0.5 g/L), were applied as spray solutions on the plants following the recommended rates that were indicated on the product labels by the manufacturing companies. Untreated-control plants were sprayed with water.

### 2.2. Vis/NIR Spectroscopy

A field-portable spectroradiometer (PSR + 3500 Spectral Evolution, Haverhill, MA, USA) was used in order to capture the reflectance spectra of the lettuce leaves. One hundred scans were acquired per pesticide treatment at the leaf top, using a replacement leaf clip, which captured the reflectance of leaf tissue at the 420–2500 nm region of the electromagnetic spectrum. Reflectance data was captured on leaf samples until harvest on the 10th day upon pesticide application, as well as after 4 and 8 days during post-harvest storage at 5 °C. A white plate (spectralon diffuse reflectance standard) was used as a standard, providing 100% reflectance in the 350–2500 nm range.

### 2.3. Antioxidant Enzyme Activities

Plant tissue was mixed with 50 mM phosphate buffer (pH 7.8) that was prepared with 0.1 mM ethylenediaminetetraacetic acid and 2% polyvinylpyrrolidone. The homogenate was centrifuged and filtered before being used to determine the antioxidant enzymes’ activities.

For POD and CAT activities, the method of Kato and Shimizu (1987) was used [26]. The POD reaction solution consisted of 50 mM phosphate buffer (pH 6.5), 20 mM guaiacol, and 200 μL of extract solution. The change in the absorbance at 470 nm was recorded in a spectrophotometer upon the addition of 40 mM H_2_O_2_. The activity of CAT was determined by measuring the rate of H_2_O_2_ reduction. The reaction solution (2.1 mL) consisted of 50 mM phosphate buffer (pH 7.0) and 2 μL enzyme extract solution and was initiated by adding 5 mM H_2_O_2_. The absorbance of the reaction solution at 240 nm was recorded every 5 s.

The APX enzyme activity was determined following the method of Nakano and Asada (1981) [27], as modified by Kato and Shimizu (1987) [26]: the final solution consisted of 50 mM potassium phosphate buffer (pH 7.0), 0.5 mM ascorbate, 0.1 mM EDTA, 1 mM H_2_O_2_, and 200 μL enzyme extract solution. The decrease in ascorbate was followed at 24 °C as a decline in absorbance at 290 nm in a spectrophotometer.

All enzyme activities were expressed as units 100 g^−1^ FW, where one unit of enzyme was defined as a change in the absorbance of 0.001 units min^−1^.

### 2.4. Lipid Peroxidation

Lipid peroxidation was assessed by determining the MDA content as described by Heath and Packer (1968) [28]. The same extract as above was heated for 20 min in a boiling-water bath with 2.5 mL 0.5% TBA in 20% trichloroacetic acid (TCA). Upon cooling, the mixture was centrifuged at 3000× *g*, and the MDA equivalent was calculated from the difference in absorbance at 532 and 600 nm. The final content of MDA was expressed in μmole per gram fresh weight (μmole g^−1^ FW)

### 2.5. Statistical Analysis

The partial least squares discriminant analysis (PLSDA) classification algorithm was implemented in this study in order to classify pesticide-free and pesticide-treated baby lettuce leaves. PLSDA is a linear classification method that combines regression algorithms and discrimination techniques, minimizing the cross-validation error [29]. The number of latent variables were selected based on the lowest root mean square cross-validation error (RMSECV) using the ‘random subsets’ method.

Moreover, advanced feature-extraction methods such as the genetic algorithm and the VIP scores were adapted, in order to detect specific regions as well as individual wavelengths of the 340–2500 nm spectrum that exhibit the most significant effect in the discriminant analysis models. The VIP scores are related to the importance of each variable in a projection model using the principal component (PCA) and partial least square (PLSA) analysis algorithms [30].

Data analyses were carried out using Microsoft Excel 2016, SPSS v. 25, MATLAB (Version R2017, The Math-works Inc., Natick, MA, USA) and PLS Toolbox 8.6 (Eigenvector Research Inc., Manson, WA, USA).

One way analysis of variance (ANOVA) was performed to estimate the effects of pesticides on biochemical components during storage, and means were separated by the least significant difference (LSD) at the 0.05 level.

## 3. Results

**Figure 1:**
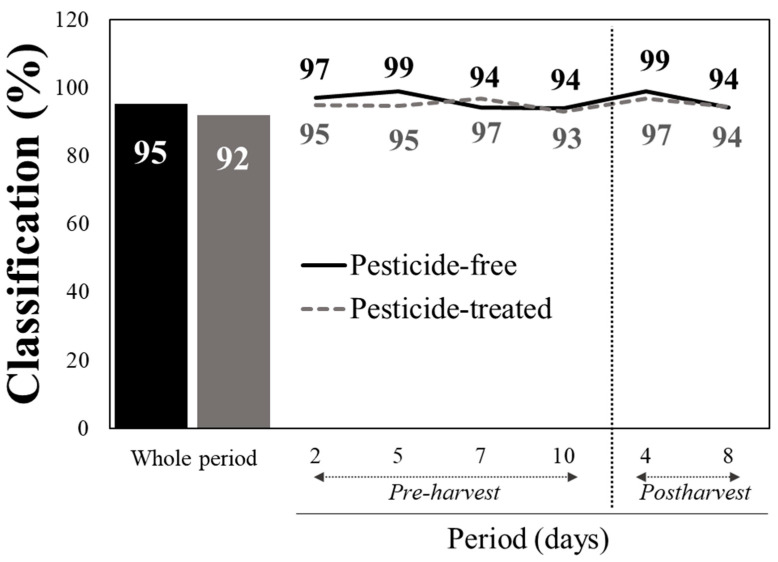
Classification rate (%) of pesticide-free and pesticide-treated baby lettuce leaves based on reflectance spectra data (340–2500 nm) within each day of pre-harvest production or postharvet storage, as well as average means for the whole period upon pooling the data of all individual days.

The Vis/NIR spectral data were initially pre-processed using standard normal variate (SNV) and second derivative techniques, before being used in the discrimination analysis of the baby lettuce leaves. The overall cross-validated pesticide-free (PF) and pesticide-treated (PT) leaves as means for the whole period upon pooling the reflectance vis/NIR data of all pre- and post-harvest measurements, using the PLSDA classifier and using 10 latent variables, were 95 and 92%, respectively, (LVs) (Figure 1). When the PLSDA algorithm was tested on the measurements within each individual day, the classification rates between PF and PT leaves were substantially improved. Indeed, starting from the second day upon the application of the fungicides and insecticides, the discrimination of the treated against the untreated plants was distinct, with the classification scores being as high as 94–97% and 93–97% for pesticide-free or treated lettuce, respectively (Figure 1). This finding is of paramount importance because it implies that more than 94 out of 100 and more than 372 out of 400 baby leaf lettuce samples were predicted as true positive, meaning they were correctly classified. Similarly, during the post-harvest period, the PF and PT samples were detected as reliably as in the pre-harvest period (94–99% and 94–97%, respectively) (Figure 1).

**Figure 2:**
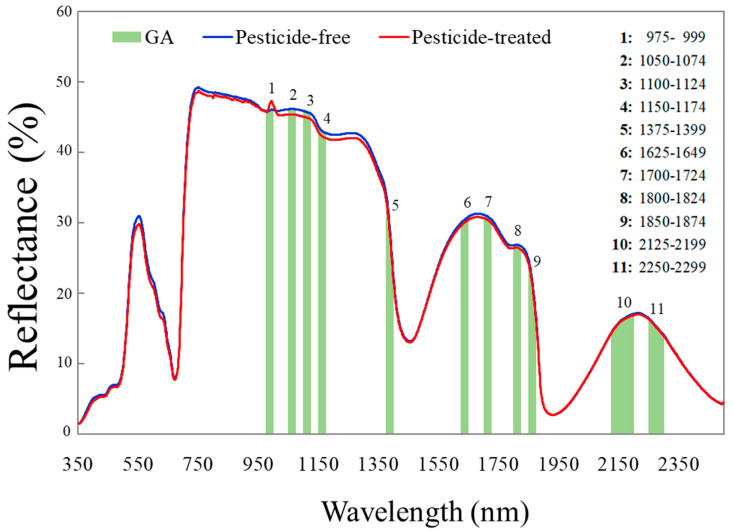
Spectra reflectance (%) of pesticide-free (blue line) and pesticide-treated (red line) baby lettuce leaves in the vis-NIR part (340–2500 nm) as average means for the whole period upon pooling the data captured in all individual days. The eleven green areas represent the parts of the spectrum that exhibited the most significant effect on the partial least squares discrimination analysis classifier and were detected using the genetic algorithm (GA).

Spectroscopy generates a large amount of data, which require sophisticated software and an advanced statistical background. The genetic algorithm was tested, and it effectively identified eleven distinct regions of the electromagnetic spectrum (975–999, 1050–1074, 1100–1124, 1150–1174, 1375–1399, 1625–1649, 1700–1724, 1800–1824, 1850–1874, 2125–2199, and 2250–2299 nm) that are able to discriminate the pesticide-free and pesticide-treated lettuce leaves (Figure 2).

**Figure 3:**
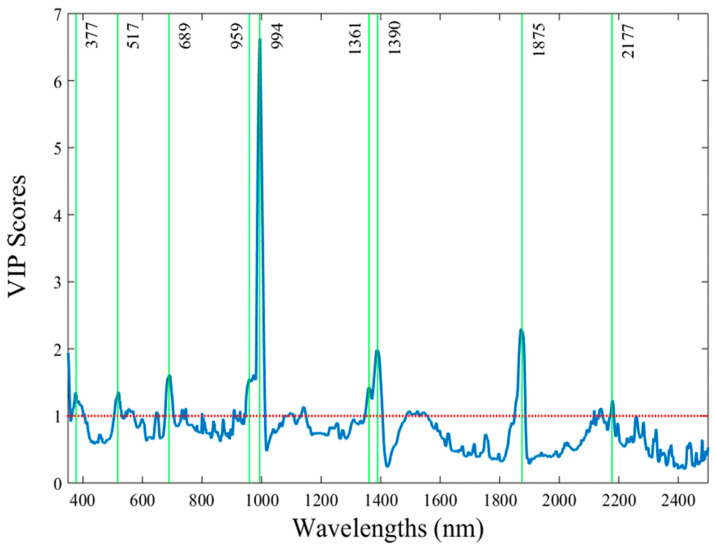
The variable importance in projection scores (VIP) in the vis/NIR part (340–2500 nm), which represents the individual effect of each wavelength on the partial least squares discrimination analysis classifier. The vertical green lines correspond to the wavelengths with the highest VIP scores. The red dot line corresponds to the lowest limit above which a wavelength exhibits a significant effect in the discriminant analysis algorithm.

**Figure 4:**
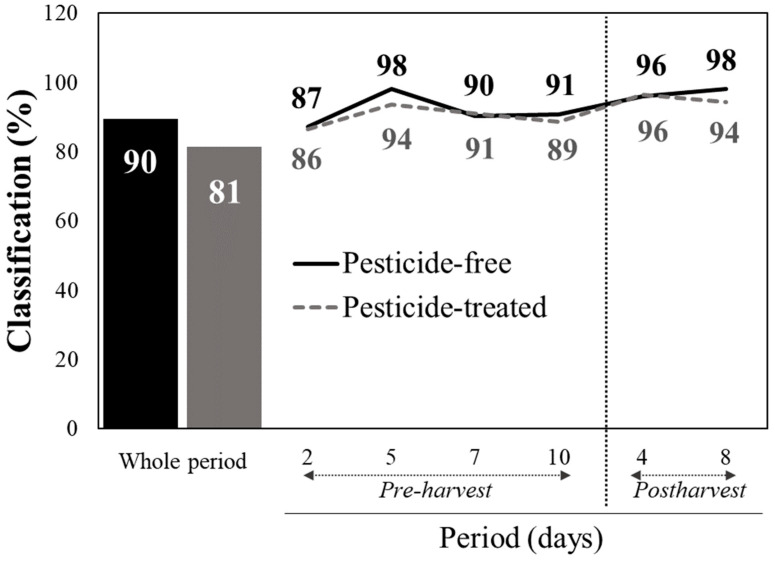
Classification rate (%) of pesticide-free and pesticide-treated baby lettuce leaves based on the reflectance spectra data at 377, 517, 689, 959, 994, 1361, 1390, 1875, and 2177 nm that were selected using the VIP scores analysis, within each day of pre-harvest production or post-harvest storage, as well as average for the whole period upon pooling the data captured in all individual days.

In order to further minimize the complexity of processing the data that were captured in the extended visible/near infrared part (340–2500 nm) by identifying the specific wavelengths that exhibit the maximum importance in the classification model, the VIP scores test was used. As a result, nine out of the total 995 wavelengths were detected (377, 517, 689, 959, 994, 1361, 1390, 1875, and 2177 nm), highlighting their unique contribution in the prediction algorithms (Figure 3). Generating discrimination models based on these VIP scores resulted in accurate detection rates of the pesticide-treated tissue, similarly to the results when all the 995 spectral reflectance data from each measurement were integrated in the analyses (Figure 4). In particular, 90% of the CVPF and 81% of the CVPT were correctly classified when the data of the whole period were integrated in the model (Figure 4). Within the pre- and post-harvest periods, the discrimination scores were still higher than 86–87% in both periods.

Notably, interesting results regarding the biochemical plant condition were also found in the study, confirming that the pesticide spray application induced a significant effect on the antioxidant mechanism of lettuce plants. In particular, the MDA content, which is an important indicator of plant stress, was significantly higher (0.546–0.563 μmole g^−1^ FW) in the leaves of pesticide-treated plants than in the untreated ones (0.385–0.393 μmole g^−1^ FW) after both 4 and 8 days of storage. The other biochemical components of the antioxidant mechanism in the plants, such as H_2_O_2_, APX, CAT, and POD, did not differ significantly between the pesticide-free and the treated leaf tissue (Table 1).

## 4. Discussion

The results of this study highlight the effectiveness of visible/near-infrared (vis/NIR) spectroscopy coupled with chemometrics as a non-destructive tool for detecting pesticide residues in baby lettuce leaves during both the production period and post-harvest storage. The high classification accuracy observed in this study—reaching up to 99% for pesticide-free (PF) and 97% for pesticide-treated (PT) samples—demonstrates the robustness of vis/NIR spectroscopy in distinguishing treated from untreated lettuce leaves. These findings are consistent with previous studies that have successfully applied vis/NIR spectroscopy to detect pesticide residues in various fruits and vegetables [9]. For instance, Sun et al. (2018) [17] achieved comparable accuracy using near-infrared transmission spectroscopy on lettuce leaves, while Zhang et al. (2023) [16] validated similar methods on cauliflowers, further demonstrating the adaptability of vis/NIR approaches to diverse agricultural products.

The pre-processing techniques employed, including standard normal variate (SNV) and second derivative, were critical in improving the value of the spectral data, thereby increasing the discrimination efficiency of the partial least squares discriminant analysis (PLSDA) classifier. The use of a genetic algorithm to identify specific regions of the electromagnetic spectrum that most significantly contributed to the classification model further underscores the potential of vis/NIR spectroscopy for precise and targeted analysis.

The identified regions, notably between 975 and 2299 nm, are beyond the visible part of the spectrum, indicating that vis/NIR spectroscopy can detect chemical changes in the plant tissues while no visual evidence existed on the tissue, and therefore the human eye would not perceive any differences. These results align with findings in studies on pakchoi [12], broccoli [15], and purple cabbage [13], where the high moisture content and specific cellular structures enhanced spectral absorption properties. Yu et al. (2021) [14] similarly reported that combining vis/NIR spectroscopy with deep learning algorithms improved residue detection in Hami melon. The consistent identification of critical spectral regions across studies reinforces the versatility of vis/NIR spectroscopy in agricultural monitoring, suggesting its potential for broader application in residue detection for various crops.

The application of the variable importance in projection scores to reduce the complexity of the spectral data while maintaining high classification accuracy (90% for PF and 81% for PT when integrating data over the entire period) is even more important. This approach simplifies the analysis by reducing the size of the data to be processed and also enhances the practical applicability of vis/NIR spectroscopy establishing the first step to develop a tool that will facilitate accurate predictions in real-world scenarios where rapid and reliable detection of pesticide residues is essential, while minimizing the complexity of the process. By narrowing the analysis to nine key wavelengths, the approach balances analytical precision with practical feasibility, a necessary step for field deployment. This methodology is in agreement with advances reported by Nazarloo et al. (2021) [11], who emphasized the importance of streamlining spectral data processing to enhance practical usability without sacrificing accuracy.

Beyond the spectral analysis, the biochemical findings provide a deeper understanding of plant stress responses to pesticide application. The significantly higher MDA content in pesticide-treated plants is a clear indicator of lipid peroxidation and oxidative stress. This result corroborates findings by Shakir et al. (2018) [31] and Homayoonzadeh et al. (2020) [32] who observed similar oxidative responses in tomato and cucumber seedlings under pesticide treatment. However, the lack of significant changes in other antioxidant markers, such as CAT, POD, and APX, suggests that the oxidative stress induced by pesticides may not uniformly activate all defense mechanisms.

Apparently, while vis/NIR spectroscopy effectively detects pesticide residues, its capability to monitor the overall biochemical stress responses in lettuce may be more limited or require additional parameters to capture these effects comprehensively. While MDA levels were captured effectively through vis/NIR spectroscopy, further studies could explore integrating additional biochemical parameters into the spectral models. Combining biochemical markers with spectral data could enhance the specificity of vis/NIR spectroscopy, providing a more comprehensive tool for assessing both residue levels and plant health.

Traditional methods, such as gas chromatography (GC) and liquid chromatography (LC) coupled with mass spectrometry (MS), remain indispensable for confirming pesticide residues due to their sensitivity and specificity. However, their destructive nature, cost, and time-intensive procedures limit their scalability for routine monitoring. Non-destructive methods like vis/NIR spectroscopy, on the other hand, offer rapid, cost-effective, and reliable alternatives. Similar trends have been observed in studies utilizing hyperspectral imaging [18], laser-induced breakdown spectroscopy (LIBS) [24,25], and surface-enhanced Raman spectroscopy (SERS) [21,22]. For example, Jiang et al. (2017) [18] used hyperspectral imaging to visualize pesticide residue distribution on mulberry leaves, demonstrating the potential of integrating vis/NIR spectroscopy with imaging techniques for enhanced residue detection.

Additionally, integrating vis/NIR spectroscopy with chemometric and machine learning tools, as demonstrated in this study, aligns with the broader shift towards precision agriculture. Advanced models, such as deep learning or convolutional neural networks, could further enhance predictive accuracy, as evidenced by recent studies on Hami melon [14] and grapes [19]. Developing portable, field-ready devices that leverage these advances could revolutionize on-site monitoring, ensuring food safety in real-time while reducing reliance on centralized laboratory analysis.

The findings from this study also contribute to the growing body of evidence supporting the necessity of pesticides in modern agriculture. Pesticides are crucial in protecting crops from pests and diseases, thereby ensuring high yields and food security [5]. However, the presence of pesticide residues on crops, particularly those consumed raw like baby leaf vegetables, necessitates the development of reliable detection methods to ensure consumer safety.

In baby leaf vegetables, which are particularly vulnerable to post-harvest spoilage due to wounding and high respiration rates [8], the ability to monitor pesticide residues alongside indicators of oxidative stress offers dual benefits. It ensures compliance with food safety standards while simultaneously providing insights into product quality during storage. These dual applications could streamline quality assurance protocols in the fresh produce industry, enhancing both efficiency and consumer trust.

Furthermore, as consumer demand for pesticide-free and organic produce continues to grow, non-destructive methods like vis/NIR spectroscopy could play a pivotal role in supporting certification processes. By providing rapid and reliable assessments, these methods could help producers differentiate their products in increasingly competitive markets.

While this study demonstrates the efficacy of vis/NIR spectroscopy, several avenues for future research remain. Future research should focus on further refining the chemometric models used in vis/NIR spectroscopy to improve the detection of a broader range of biochemical responses to pesticide treatment. Integrating this method with complementary techniques, such as hyperspectral imaging or Raman spectroscopy, could provide a more holistic approach to residue detection. Additionally, extending this research to other crops and environmental conditions could validate its robustness and scalability. Finally, exploring the potential of vis/NIR spectroscopy for monitoring other chemical contaminants or nutritional attributes could further broaden its applications in plant health monitoring [33,34].

## 5. Conclusions

This study demonstrates the effectiveness of vis/NIR spectroscopy coupled with chemometrics in rapidly and accurately detecting pesticide residues in lettuce baby leaves. The high classification accuracy, combined with the ability to monitor oxidative stress markers like MDA, underscores the potential of this non-destructive method in ensuring the safety and quality of fresh produce. As the demand for safe and high-quality food continues to grow, vis/NIR spectroscopy offers a valuable tool for the agricultural industry, contributing to the sustainable production of pesticide-treated crops while safeguarding consumer health.

## Figures and Tables

**Table 1:** Malondialdehyde (MDA) and hyperoxide (H_2_O_2_) content, as well as peroxidase (POD), catalase (CAT), and ascorbate peroxidase (APX) activity, of baby leaf lettuce during post-harvest storage for 4 and 8 days at 5 °C in perforated plastic bags (*n* = 3).

	MDA Content		H_2_O_2_ Content		POD Activity		CAT Activity		APX Activity	
	(μmole g^−1^ FW)				(units mg^−1^ Protein)					
	4 d	8 d	4 d	8 d	4 d	8 d	4 d	8 d	4 d	8 d
Pesticide-free	0.393 ^x^ b ^y^	0.385 b	0.624	0.568	0.042	0.025	0.176	0.207	0.018	0.022
Pesticide-treated	0.546 a	0.563 a	0.703	0.550	0.037	0.030	0.195	0.188	0.021	0.020
*p*	0.009	<0.001	ns ^W^	ns	ns	ns	ns	ns	ns	ns

^x^ each number is the average of 3 replications. ^y^ different letters within the same column indicate significant differences according to the LSD test. ^W^ ns: no significant differences between the means in the same column.

## Data Availability

Data is available upon request to the authors.

## References

[1] Sarma U., Bhavya T.R. (2024). Dietary phytonutrients in common green leafy vegetables and the significant role of processing techniques on spinach: A review. Food Prod. Process. Nutr..

[2] Song J., Huang H., Hao Y., Song S., Zhang Y., Su W., Liu H. (2020). Nutritional quality, mineral and antioxidant content in lettuce affected by interaction of light intensity and nutrient solution concentration. Sci. Rep..

[3] Ahmad M.F., Ahmad F.A., Alsayegh A.A., Zeyaullah M., AlShahrani A.M., Muzammil K., Saati A.A., Wahad S., Elbendary E.Y., Kambal N. (2024). Pesticides impacts on human health and the environment with their mechanisms of action and possible countermeasures. Heliyon.

[4] Sindhu S., Manickavasagan A. (2023). Nondestructive testing methods for pesticide residue in food commodities: A review. Compr. Rev. Food Sci. Food Saf..

[5] Popp J., Pető K., Nagy J. (2013). Pesticide productivity and food security. A review. Agron. Sustain. Dev..

[6] Chen W., Long F., Song G., Chen J., Peng S., Li P. (2020). Rapid and sensitive detection of pesticide residues using dynamic surface-enhanced Raman spectroscopy. J. Raman Spectrosc..

[7] Sánchez F.G., Blanco C.C. (1988). Spectrofluorometric Determination of Pesticide Residue Mixtures by Isodifferential Derivative Spectroscopy. Anal. Chem..

[8] Saltveit M.E. (2000). Wound induced changes in phenolic metabolism and tissue browning are altered by heat shock. Postharvest Biol. Technol..

[9] Jamshidi B., Mohajerani E., Jamshidi J., Minaei S., Sharifi A. (2015). Non-destructive detection of pesticide residues in cucumber using visible/near-infrared spectroscopy. Food Addit. Contam. Part A.

[10] Tsagkaris A.S., Pulkrabova J., Hajslova J. (2021). Optical screening methods for pesticide residue detection in food matrices: Advances and emerging analytical trends. Foods.

[11] Nazarloo A.S., Sharabiani V.R., Gilandeh Y.A., Taghinezhad E., Szymanek M. (2021). Evaluation of different models for non-destructive detection of tomato pesticide residues based on near-infrared spectroscopy. Sensors.

[12] Li M., Zhang X., Jiang Q. (2018). Qualitative Identification of Pesticide Residues in Pakchoi Based on Near Infrared Spectroscopy. IOP Conf. Ser. Mater. Sci. Eng..

[13] Li M., Lu L., Zhang X. (2021). Qualitative Determination of Pesticide Residues in Purple Cabbage Based on near Infrared Spectroscopy. J. Phys. Conf. Ser..

[14] Yu G., Ma B., Chen J., Li X., Li Y., Li C. (2021). Nondestructive identification of pesticide residues on the Hami melon surface using deep feature fusion by Vis/NIR spectroscopy and 1D-CNN. J. Food Process Eng..

[15] Nitta Y., Ishizawa H., Goto T., Miyahara Y., Komatus K. A rapid measurement of pesticides residues in broccoli based on IR spectroscopy. Proceedings of the SICE-ICASE International Joint Conference, ICASE 2006.

[16] Zhang M., Xue J., Li Y., Yin J., Liu Y., Wang K., Li Z. (2023). Non-destructive detection and recognition of pesticide residue levels on cauliflowers using visible/near-infrared spectroscopy combined with chemometrics. J. Food Sci..

[17] Sun J., Ge X., Wu X., Dai C., Yang N. (2018). Identification of pesticide residues in lettuce leaves based on near infrared transmission spectroscopy. J. Food Process Eng..

[18] Jiang S., Sun J., Xin Z., Mao H., Wu X., Li Q. (2016). Visualizing distribution of pesticide residues in mulberry leaves using NIR hyperspectral imaging. J. Food Process Eng..

[19] Ye W., Yan T., Zhang C., Duan L., Chen W., Song H., Zhang Y., Xu W., Gao P. (2022). Detection of Pesticide Residue Level in Grape Using Hyperspectral Imaging with Machine Learning. Foods.

[20] Li Y., Sun Y., Peng Y., Dhakal S., Chao K., Liu Q. Rapid detection of pesticide residue in apple based on Raman spectroscopy. Proceedings of the Sensing for Agriculture and Food Quality and Safety IV.

[21] Pham U.T., Phan Q.H.T., Nguyen L.P., Luu P.D., Doan T.D., Trinh H.T., Dinh C.T., Nquyen T.V., Tran T.Q., Le D.X. (2022). Rapid Quantitative Determination of Multiple Pesticide Residues in Mango Fruits by Surface-Enhanced Raman Spectroscopy. Processes.

[22] Ma P., Wang L., Xu L., Li J., Zhang X., Chen H. (2020). Rapid quantitative determination of chlorpyrifos pesticide residues in tomatoes by surface-enhanced Raman spectroscopy. Eur. Food Res. Technol..

[23] Tognaccini L., Ricci M., Gellini C., Feis A., Smulevich G., Becucci M. (2019). Surface enhanced raman spectroscopy for in-field detection of pesticides: A test on dimethoate residues in water and on olive leaves. Molecules.

[24] Du X., Dong D., Zhao X., Jiao L., Han P., Lang Y. (2015). Detection of pesticide residues on fruit surfaces using laser induced breakdown spectroscopy. RSC Adv..

[25] Liu L., Wang Y., Gao C., Huan H., Zhao B., Yan L. (2014). Photoacoustic Spectroscopy as a Non-destructive Tool for Quantification of Pesticide Residue in Apple Cuticle. Int. J. Thermophys..

[26] Kato M., Shimizu S. (1985). Chlorophyll metabolism in higher plants. VI. Involvement of peroxidase in chlorophyll degradation. Plant Cell Physiol..

[27] Nakano Y., Asada K. (1981). Hydrogen peroxide is scavenged by ascorbate-specific peroxidase in spinach chloroplasts. Plant Cell Physiol..

[28] Heath R.L., Packer L. (1968). Photoperoxidation in isolated chloroplasts: I. Kinetics and stoichiometry of fatty acid peroxidation. Arch. Biochem. Biophys..

[29] Ballabio D., Consonni V. (2013). Classification tools in chemistry. Part 1: Linear models. PLS-DA Anal. Methods.

[30] Wold S., Sjöström M., Eriksson L. (2001). PLS-regression: A basic tool of chemometrics. Chemom. Intell. Lab. Syst..

[31] Shakir S.K., Irfan S., Akhtar B., Rehman S., Daud M.K., Taimur N., Azizullah A. (2018). Pesticide-induced oxidative stress and antioxidant responses in tomato (*Solanum lycopersicum*) seedlings. Ecotoxicology.

[32] Homayoonzadeh M., Moeini P., Talebi K., Roessner U., Hosseininaveh V. (2020). Antioxidant system status of cucumber plants under pesticides treatment. Acta Physiol. Plant.

[33] Sun J., Cong S., Mao H., Wu X., Yang N. (2017). Quantitative detection of mixed pesticide residue of lettuce leaves based on hyperspectral technique. J. Food Process Eng..

[34] Dhakal S., Peng Y., Li Y., Chao K., Qin J., Zhang L., Xu T. Rapid detection of chlorpyrifos pesticide residue concentration in agro-product using Raman spectroscopy. Proceedings of the Sensing for Agriculture and Food Quality and Safety IV.

